# Health-related Quality of Life and Domain-specific Associated Factors among Patients with Type2 Diabetes Mellitus in South India

**DOI:** 10.1900/RDS.2022.18.34

**Published:** 2022-03-31

**Authors:** Jansirani Natarajan, Sheila Mokoboto-Zwane

**Affiliations:** 1 fundamentals and Administration Department, College of Nursing, Sultan Qaboos University, Muscat Oman; 2 College of Human Sciences, Department of Health Studies, University of South Africa.

**Keywords:** health-related quality of life, type 2 diabetes mellitus, south India, perception, predictors

## Abstract

**OBJECTIVE:**

Type 2 diabetes mellitus (T2DM) is a chronic metabolic disorder that has a major impact on health-related quality of life (HRQOL). The economic burden of the disease, along with its complications, negatively impact the individual, family, and society of Indian diabetic patients. This study explored the perception of the diabetic HRQOL of South Indian type2diabetic patients.

**METHODS:**

This study was a cross-sectional descriptive quantitative study conducted in a tertiary care hospital in Chennai, Tamil Nadu, South India. Using the simple random sampling technique, we collected data from 352 T2DM patients aged ≥ 30 years of age who were diagnosed for a minimum of one year. Data collection occurred from June to August 2017. Data were analysed using IBM SPSS, Version 22.

**RESULTS:**

Overall, 90% of patients with T2DM perceived poor HRQOL. The total and the domain- specific mean scores of HRQOL were high indicating poor HRQOL in energy mobility, diabetes control, anxiety and worry, social burden, and sexual functioning domains. Being female, increasing age, lower education levels, lower family income, and uncontrolled fasting blood glucose levels predicted poor HRQOL of patients with T2DM.

**CONCLUSIONS:**

T2DM impacted the HRQOL in all measured domains of participants. A patient-centred approach to diabetes management can be incorporated to improve or enhance the health-related quality of patients’ lives. Improved HRQOL also may lead to fewer hospitalizations, and hence, reduce healthcare costs.

## 1 Introduction

Type 2 diabetes mellitus (T2DM) is a common and growing health problem worldwide, especially in middle and low-income countries. Diabetes mellitus (DM) is a group of metabolic disorders in which blood sugar levels are high over a prolonged period and is due to either the pancreas not producing enough insulin or the cells of the body not responding properly to the insulin produced. There are 415 million people worldwide who currently experience DM and 78.3 million are from Southeast Asia [1]. The prevalence of DM globally is predicted to increase to 629 million by 2045 without interventions to halt the increase in the disease [2].

In 2013, the prevalence of DM in India was closer to the world average (9.1% vs 8.3%) [3]. T2DM is more prevalent in the southern part of the country, compared to the northern and eastern parts [4]. One study estimated the prevalence of DM in 2016 to be 21.9% in the city of Chennai [5]. It was estimated that 2.2 billion USD are needed to treat all the patients with T2DM in India [6]. T2DM is a major cause of heart disease and stroke, as well as the leading cause of kidney failure and new cases of blindness among adults in India [7]. T2DM has a significant impact on the lives of patients, their families, and the healthcare system. The chronicity of the disease and the potential for serious consequences often result in a significant financial burden and a decreased quality of life [8]. It is necessary to explore T2DM patients’ perspectives to understand the impact of this deadly disease on the health-related quality of life (HRQOL) so that adaptive strategies can be developed.

T2DM is a chronic non-communicable disease (NCD) that can lead to many complications, possibly resulting in disability. Complications of T2DM include micro vascular (nephropathy, retinopathy, and neuropathy) and macro-vascular (cardiovascular diseases, cerebrovascular accident, and diabetic foot) complications; with comorbidities, it can cause a substantial decrease in the patients’ quality of life [9]. Another sad reality of Indian ethnicity is their genetic predisposition to develop diabetes. As Indian diabetic patients delay in seeking medical help, complications are on the rise, worsening the economic burden on the individual and society. These reasons can lead to poor HRQOL among diabetes patients which can affect their compliance to the therapeutic regimen and their coping with the disease. HRQOL is a multidimensional concept that explores the perception of the physical as well as mental health of the patients suffering from chronic diseases [7]. The chronic nature of T2DM with its complications can pose a burden of the disease to the individual, family, and society. Thus, any healthcare provider aims to address the issues and difficulties these patients are experiencing physically, mentally, and socially and to develop ways of improving their HRQOL. T2DM, both due to its chronic nature and multisystem involvement, seriously affects the HRQOL of the patient, and to an extent, that of others as well.

One study shows that the total prevalence of diabetes is chiefly contributed by the urban population in Tamil Nadu and the prevalence of T2DM in 2016 is estimated to be 21.9% in the city of Chennai [5]. Diabetes complications, comorbidities, and cost of treatment affect the quality of life of an affected individual. Diabetes demands better care and control. With emerging advancements in medicine, the longevity of life is assured. However, the heavy economic burden of T2DM is borne by the individuals and families in India without proper universal health coverage.

HRQOL assessment is considered an important measure of outcome in diabetes, as understanding the personal views, judgments, and preferences of patients that influence their perception can guide us in developing management strategies to improve their HRQOL. An Indian study on T2DM patients revealed that patients who could adjust emotionally to the disease experienced a greater sense of well-being [10]. Studies show that psychological problems experienced by diabetes patients adversely affected their regimen adherence, and eventually their HRQOL [11]. The Diabetes Attitudes Wishes and Needs’ (DAWN) study conducted across 17 countries, including India, reported that nearly 52% of Indian T2DM patients reported diabetes distress and 27% experienced diabetes-related discrimination. The authors also emphasised the importance of patient-centred and family-supported care for improving the HRQOL of type2 diabetes patients [12].

India currently faces an uncertain future concerning the potential burden that diabetes may impose for the country. Several studies demonstrate that T2DM has a strong negative impact on HRQOL, especially in the presence of complications. However, most of the studies have been conducted in developed countries and studies in developing countries are rare. Preliminary findings from the northern part of India have reported poor HRQOL of T2DM patients [13]. HRQOL issues are important because they predict an individual’s capacity to manage his/her disease and maintain long-term health and well-being. Therefore, it is imperative to assess the perception of the HRQOL of T2DM patients to identify and implement strategies that address the health needs of South Indian T2DM patients. This study is the first of its kind to explore the perception of South Indian T2DM on their HRQOL.

## 2 Methods

### 2.1 Study design, setting and sample

This study was conducted using a quantitative, cross-sectional, and descriptive research design to elicit the information from the perspectives of diabetic people on their HRQOL and to identify the predicting factors of their HRQOL. Cohen’s formula was used to calculate the sample size and data were collected from 352 T2DM patients. The participants were included if they were aged > 30 years, in treatment for diabetes for at least one year, and possessed sound mental health. Participants attending the diabetic outpatient department of Dr Hariharan’s Institute of Diabetes in Chennai city were included in the study.

### 2.2 Data collection procedure

Type 2 diabetic patients were listed down from the hospital registers and using a simple random sampling technique, every fifth patient was selected. They were approached by two trained female research assistants in the outpatient department (OPD) of the hospital. Two days of training were given to the research assistants about study purpose, interview techniques, using the questionnaire, and ethical aspects of the study. After obtaining informed consent from the participants to ensure voluntary participation in the study, they were taken to a private room allotted for interviews. These diabetes patients follow the routine of the hospital—they come early in the morning, give blood following fasting, eat, and wait for two hours before giving post-prandial blood for testing. While they were waiting to meet the diabetologist for their regular follow-up, they were approached for data collection. After obtaining informed consent, we distributed self-report questionnaires to them.Demographic characteristics and clinical profile of the participants were collected followed by theDiabetes-39 Quality of Life Questionnaire. This tool uses a visual analogue scale ranging from 1 to 7, explaining “not affected at all” (1-4) to “extremely affected” (5-7). As the total score increases, it indicates the lower levels of HRQOL. Studies have reported it to be a reliable scale with a Cronbach’s α score of 0.917 [14]. The instrument consisted of 39 items, covering five dimensions (domains) of the health-related quality of life of people with DM — energy and mobility (15 items), diabetes control (12 items), anxiety and worry (4 items), social impact (5 items), and sexual behaviour (3 items). Permission to use this scale was received from the author. Information was recorded by the interviewer in the specified formats. Anonymity and confidentiality were maintained, as only numbers were used to identify participants.

### 2.3 Data analysis

Data were tested for normal distribution before being analysed, using the IBM SPSS Version 22, and was found to be normally distributed. The internal consistency of the responses from the participants was good with a Cronbach’s α of 0.931. The data were summarized using frequencies and percentages. The relationship between variables was determined using chi-square and the predictors of diabetes HRQOL was analysed with multiple regression and all the tests were considered statistically significant at p<0.05.

## 3 Results

As **Table1** shows, the average age in years of the patients was 58.68±11.48. Overall, 152 (43.2 %) of the patients were males and 200 (56.8 %) were females. The highest level of education attained by more than half of the patients (62.8 %) was elementary school or some secondary education. The majority (88.6 %) of the patients were married and more than three-fourths (79.8%) of the patients lived within the urban area; half of the patients (50.6%) had a monthly income of more than 10,000 INR, while the other half reported an income of less than 10,000 INR. A majority (60.8%) of patients were non-vegetarians. Most (73%) of them were living in their own house. A majority (35.2 %) of them had fasting blood sugar levels between 111 and 140 mmol/l, 24.4% of them had between 141 and 200 mmol/l, and 24.4% of them had fasting blood sugar levels above 200 mmol/l.

**Table 1.** Socio-demographic and clinical characteristics of the participants, N=352

SOCIO-DEMOGRAPHIC CHARACTERISTICS		Frequency	%
GENDER	Male	152	43.2
	Female	200	**56.8**
AGE	Mean=58.68, Sd=11.480		
PLACE OF RESIDENCE	Urban	281	**79.8**
	Semi-Urban	52	14.8
	Rural	19	5.4
EDUCATIONAL LEVEL	Illiterate	25	7.1
	Elementary School(Until 8th Grade)	127	**36.1**
	High School(Grades 9&10)	59	16.8
	Higher Secondary(Grades 11&12)	35	9.9
	University	106	30.1
MARITAL STATUS	Unmarried	11	3.1
	Married	312	**88.6**
	Separated/ Divorced	8	2.3
	Widowed	21	6.0
MONTHLY INCOME	Less Than 10000 Inr	174	49.4
	10000inr And Above	178	**50.6**
FOOD HABITS	Vegetarian	138	39.2
	Non-Vegetarian	214	**60.8**
HOUSING STATUS	Own House	256	**72.7**
	Rent House	96	27.3
FASTING BLOOD SUGAR	70-110mmol/L	77	21.9
VALUES	111-140 Mmol/L	124	35.2
	141-200 Mmol/L	65	18.5
	>200 Mmol/L	86	24.4

**Figure 1.**
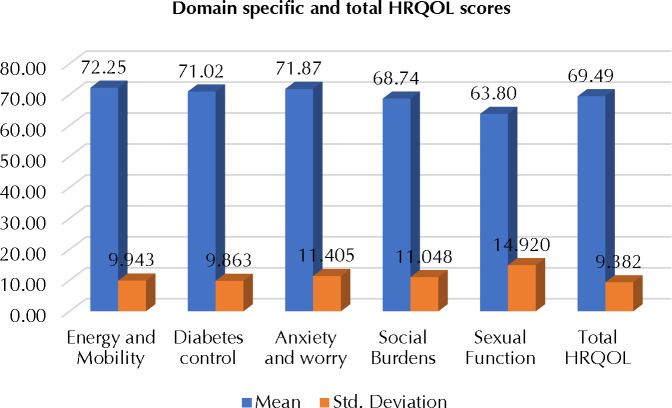
Mean and SD of total and domain-specific HRQOL of the participants

**Figure 1** projects the mean and standard deviation (SD) scores of the five domains of the D-39 questionnaire. The raw scores were converted into percentages and scores up to 57.1% indicate “not at all affected and scores above 57.1% indicates “extremely affected. “The mean and SD of the five domains of D-39 were as follows: energy and mobility — 72.25±9.943; diabetes control — 71.02±9.863; anxiety and worry — 71.87±11.405; social burden — 68.74±11.048; and sexual functioning — 63.80±14.920. The mean (± SD) of the total HRQOL scores of the participants was 69.49±9.382.

**Figure 2.**
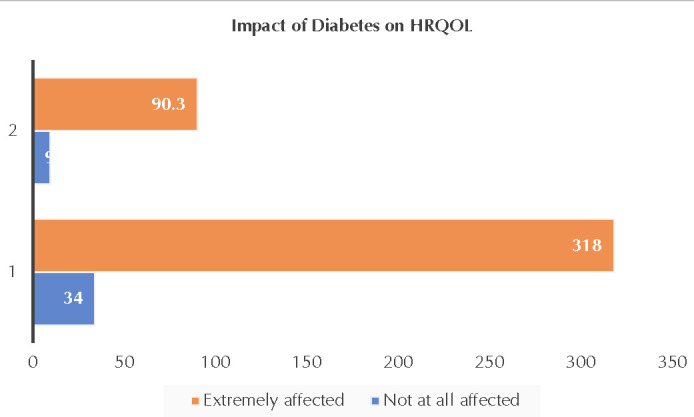
Frequency and percent of diabetes patients based on the impact on HRQOL

Based on the total HRQOL score percent, it was categorized as not at all affected if the scores were 57% and less and extremely affected if the score was above 57%. **Figure 2** depicts that the majority (90.3%) of the diabetes patients perceived to be extremely affected regarding their HRQOL.

**Table 2** revealed that there was a statistically significant difference in the perception of HRQOL as “not at all affected” and “extremely affected” among male and female diabetes patients. A higher percentage of female patients (94%) were extremely affected by diabetes than male patients (85.5%), Ӽ^2^ =7.106, p=0.008.

**Table 2.** Association between the impact of diabetes on HRQOL and gender

Gender	Impact of diabetes on Total HRQOL		Pearson chi-square value	p-value
	Not at all affected (up to 57%)	Extremely affected (57.1 to 100)		
Male	22(14.5%)	130(85.5%)		0.008	
Female	12(6%)	188(94%)	7.106	

P-value significant at <0.05 level

Results of multiple linear regression revealed that female gender, lower educational status, having uncontrolled fasting blood sugar levels, being in the older age group, and having lower family income predicted poor HRQOL of type2 diabetic patients as **Table 3** shows.

**Table 3.** Multiple linear regression model of predictors of the HRQOL of diabetes patients

Variables	Odds ratio	B	SE	Wald	P- value
Gender(1)	.353	-1.042	.478	4.740	0.029
Place of residence (urban vs Rural)	2.155	.768	.489	2.463	0.117
Educational level (below high school vs Highersecondary and above)	.539	-.617	.193	10.236	0.001
Fasting Blood Glucose levels(NORMAL VS ABOVE NORMAL)	2.319	.841	.234	12.902	0.000
Age(HIGHER VS LOWER)	1.047	.046	.019	6.202	0.013
Marital status(MARRIED VS SEPARATED)	.578	-.548	.439	1.561	0.212
Income (High vs low)		.540	.257	4.422	0.035
Food habits (vegetarian vs nonvegetarian)		.424	.414	1.052	0.305
Housing status (OWN VS RENT)		.277	.478	.336	0.562
Constant		-1.892	2.138	.783	0.376

Note:

a. Variable(s) entered on step 1: Gender, Place, Education, FBG, Age, Marital, Income, Food, and House.

## 4 Discussion

In the present study, we intended to explore and describe the perceptions of health-related quality of life of T2DM patients attending Dr. Hariharan Institute of Diabetes, Chennai, South India. HRQOL is vital in T2DM because poor HRQOL leads to reduced self-care, triggering uncontrolled blood sugar levels, an increased risk of complications, and the exacerbation of diabetes.

Mean age of 59 years for diabetes patients in this study is consistent with the World Health Organization declaration that T2DM is more common in the older generation in developing countries. We note interesting gender differences in the present study that are like ones in previous literature, where men reported significantly better HRQOL than women [15,16]. Education levels proved to be an important determinant of HRQOL, with university and higher secondary level educated patients perceiving a better HRQOL than less educated patients. One might hypothesize that diabetes, as a disease with high demands for selfmanagement, could be managed better by a group of educated patients. Similar findings were observed in a Glasgow study and other research that lower education level was associated with a lower quality of life in diabetes patients [17,18]. This finding differs from studies where education levels did not influence the HRQOL of diabetes patients [19,20]. Those who are well-educated can have a good knowledge of the disease, its management, and its effects on them. They may seek early and better management strategies for controlling diabetes and they usually belong to the upper levels of society [21]. Income is another predictor of HRQOL for diabetes patients.

Lesser income was related to lower HRQOL. Income and social status are evidenced in literature as strong predictors of HRQOL. Our study also revealed that unemployed patients and housewives had a lower HRQOL. The economic crisis in India contributes to the heavier burden on the individuals who need to pay a large amount for their treatment. This could be a topic for future research — to estimate the disease burden on the Indian economy, primarily the burden of diabetes. It is well-documented that the cost of treatment to the patient ultimately burdens both the formal healthcare system and the patient’s health, thereby affecting their HRQOL.

Our results indicate that more than half of the patients had uncontrolled blood sugar levels which were significantly associated with their poor HRQOL. This result supports the finding of earlier studies that poor HRQOL was associated with poor glycaemic control [22,23]. Our analysis of glycaemic control and diabetes-specific HRQOL may explain the symptoms of diabetes-like fatigue, vision problems, leg pain, increased thirst and hunger, frequent infections, and other associated problems caused by constantly elevated sugar levels, which in sequence, can affect their HRQOL. Specifically, our study has revealed a significantly lower HRQOL in the energy and mobility and diabetes control domains.

Our results revealed that energy and mobility, anxiety and worry, and diabetes control impact HRQOL the most, followed by social burden and sexual functioning. When considering the amplitude of the overall responses to the D-39 questionnaire, 90% of the patients have scored above 57%, suggesting “extremely affected” (5-7 in the Likert scale) and indicating poor HRQOL. Our findings are consistent with many other studies conducted among T2DM patients in developed countries as well as in India [24,25] and HRQOL diabetes patients were not affected in a study conducted among rural South-India. A higher percentage of females was affected adversely in their HRQOL compared to their male diabetic counterparts, a finding consistent with those of other investigators [26]. Culturally speaking, South Indian women cannot abandon the “caretaker role” in the family and they pay more attention to the health of the men and children, often neglecting their own wellbeing. There is a need to improve the HRQOL of women with diabetes.

The domain of energy and mobility had the highest mean score, indicating poor HRQOL of our the diabetes patients we studied, a finding consistent with ones of other researchers [24]. Medication adherence and good glycaemic control are the recommended interventions for these patients to restore their energy levels. Dietary management, focusing on a well-balanced diet and vitamin supplements can improve their health status. An exercise intervention to improve energy and mobility levels needs to be designed that is considers the capacity of individual diabetes patients.

Diabetes demands continuous care in sustaining treatment compliance, diet management, and monitoring blood sugar levels to prevent adverse complications. Diabetes self-care is conceptualised as the awareness of the illness, as well as learning ways to live with the complications [27]. The patient needs education to enhance their expertise in selfmanagement [28]. Almost all diabetes patients have perceived that their diabetes control is due to their total individual effort like diet restriction and medications, as Patel and colleagues found [20]. Their study indicated poorer HRQOL among uncontrolled diabetes patients compared to ones who controlled their diabetes.

The anxiety and worry domain had the second highest mean score among participants. Previous studies also have indicated a negative relationship between stress and HRQOL in diabetes patients [28,29,30]. Moderate to high levels of worrying may be a response to poor dietary management, diabetes complications, and poor glycaemic control [31]. Depression and associated lower HRQOL was reported among the Indian T2DM patients, compared to controls in the study. Additionally, lower HRQOL was associated with poor glycaemic control in the same study [32]. These findings have implications for the development of counselling programmes to teach diabetes care and ways to manage stress, thereby improving treatment adherence and health-promoting behaviours. Family support is also crucial in keeping diabetes under control.

Despite current advances in the management of diabetes and the dissemination of disease-specific information, there is still a propensity in Indian society to brand the person with diabetes as someone who is disadvantaged. This social stigma is frequently internalised by patients and appears to be an aspect of lower HRQOL perception. Diabetes patients are often discouraged from being involved in social events because of their dietary restrictions and insulin administration. Those perceived to be satisfied with their social relationships had a better quality of life. This could be because of the psychological effect of sharing their worries, which makes them feel reassured and joyful. Numerous studies conducted globally demonstrated that social relationships and friendships have a vital role to play in the quality of life [33,34]. Patients’ family members should be involved in counselling and health education sessions during hospital follow-up visits.

The sexual functioning domain had the lowest score among the five domains of the study. In India, sexuality is not a topic that many people can discuss openly directly discuss, and difficulties related to it remain suppressed. This could be the likely reason that several participants, especially women, hesitated in answering questions related to the sexual domain. Therefore, it should be cautiously concluded that it is not possible to be clear about the reliability of the sexual functioning domain score. This finding highlights the cultural issues affecting HRQOL assessments in diabetes patients. Diabetes centres should be furnished with sexologists for better assessment and treatment of diabetes patients’ problems.

Female gender, increasing age, lower educational status, lower income, and uncontrolled fasting blood sugar levels predicted poor HRQOL among diabetic patients in this study. Most of the published studies have presented female gender and older age to be associated with overall inferior health-related quality of life scores among persons with diabetes [35,36] and these findings are consistent with this study. Support groups for women with diabetes should be formed at the community level to motivate them towards selfmanagement.

Clinically, one can conclude that poor glycaemic control will be associated with both increased prevalence and incidence of macro vascular and microvascular complications, ultimately leading to decreased HRQOL [37]. However, studies have produced contrasting results, with some showing poor glycaemic control to be associated with poorer HRQOL [38,39] whereas others found no association. The current study predicted an association between glycaemic control and HRQOL. Measures should be designed and taught to diabetes patients to control their blood sugar levels to avoid disease-related complications. Education on self-care and self-control have a noted impact on controlling disease progression and improving HRQOL, as one meta-analysis noted [40].

The vision of the Ministry of Health of India is that by the year 2020, diabetes patients will achieve good glycaemic control resulting in improved HRQOL, which will be characterised by a reduced number of complications, comorbidities and mortality [41]. However, this vision cannot be fulfilled without the adherence to a therapeutic diabetes regimen by patients — mainly diet control, medications, exercise, and regular follow-ups. The government should focus on the challenges perceived by persons with diabetes and develop policies to improve diabetes care in the country which can improve patients’ HRQOL.

### 4.1 Recommendations

The government of India has initiated many national health programmes, drafted many policies, signed many declarations, attended many summits, made many commitments, and set many goals to realise the vision of health for all by 2020. What is needed now is the implementation and strengthening of the above initiatives by all sectors. There is a need to strengthen the multi-sectorial approach to this fast- increasing chronic disease, where each sector will play its role and the combined effort will result in the improvement of the HRQOL of diabetes patients. We recommend that a transitional care centre, involving a team of healthcare providers that can provide comprehensive supportive care for diabetes patients, be established which includes a diabetologist, diabetes nurse-educator, a dietician, a clinical psychologist, and a physiotherapist. There is a need for an awareness programme for improve self-management of diabetes, which could be of value to lower resource settings like India. Tertiary care hospitals in India should adopt this self-care management model with standards recommended by the American Diabetes Association.

### 4.2 Limitations

This study was limited to exploring the perception of overall HRQOL and domain specific HRQOL of T2DM patients in a diabetes speciality centre in Chennai, Tamil Nadu, South India; thus, our findings cannot be generalised. The researchers depended on the selfreports of diabetic patients to measure HRQOL, which may not be the actual perception of the patients. A novel sensitivity-analysis procedure for investigating and reducing the bias of self-reported surveys is suggested for use in future research.

## Conclusion

Given that the disease is now lightly visible across all sections of society in India, there is demand for the urgent implementation of the above recommendations at a “grassroots” level to contain the new-age diabetes pandemic. A patient-centred approach to diabetes management can be incorporated to improve or enhance the health-related quality of patients’ lives. Improved HRQOL also may lead to fewer hospitalisations, and hence, reduce healthcare costs. It is hoped that our findings could be useful to health professionals in developing health behaviour modification programs along with self-care management education, to improve the HRQOL of Indian T2DM patients.

## Abbreviations

T2DM: type 2 diabetes mellitus

HRQOL: health-related quality of life

DM: diabetes mellitus

NCD: non-communicable disease

DAWN: the Diabetes Attitudes Wishes and Needs

OPD: outpatient department

SD: standard deviation
